# An Appendectomy Increases the Risk of Rheumatoid Arthritis: A Five-Year Follow-Up Study

**DOI:** 10.1371/journal.pone.0126816

**Published:** 2015-05-13

**Authors:** Ya-Mei Tzeng, Li-Ting Kao, Senyeong Kao, Herng-Ching Lin, Ming-Chieh Tsai, Cha-Ze Lee

**Affiliations:** 1 Graduate Institute of Life Science, National Defense Medical Center, Taipei, Taiwan; 2 School of Public Health, National Defense Medical Center, Taipei, Taiwan; 3 Sleep Research Center, Taipei Medical University Hospital, Taipei, Taiwan; 4 Division of Gastroenterology, Department of Internal Medicine, Cathay General Hospital, Taipei, Taiwan; 5 Department of Internal Medicine, National Taiwan University Hospital, Taipei, Taiwan; Nippon Medical School Graduate School of Medicine, JAPAN

## Abstract

Many studies have reported a possible association of an appendectomy with rheumatoid arthritis (RA). However, findings of the relationship between an appendectomy and RA remain inconsistent. Furthermore, all such studies were conducted in Western societies, and relevant studies on the relationship between an appendectomy and RA in Asian countries are still lacking. In this study, we investigated the relationship between an appendectomy and the subsequent risk of RA using a population-based dataset. We retrieved data for this retrospective cohort study from the Taiwan “Longitudinal Health Insurance Database 2005”. We included 4,294 subjects who underwent an appendectomy in the study cohort and 12,882 matched subjects in the comparison cohort. We individually tracked each subject for a 5-year period from their index date to identify those who developed RA. A stratified Cox proportional hazard regression was performed to calculate the hazard ratio (HR) and its corresponding 95% confidence interval (CI) for the subsequent development of RA during the 5-year follow-up period between subjects who underwent an appendectomy and comparison subjects. Of the sampled subjects, 93 (0.54%) received a diagnosis of RA during the 5-year follow-up period: 33 from the study cohort (0.77% of subjects who underwent an appendectomy) and 60 from the comparison cohort (0.47% of comparison subjects) (*p*<0.001). After censoring individuals who died during the follow-up period and adjusting for subjects’ monthly income and geographic region, the HR of RA during the 5-year follow-up period was 1.61 (95% CI = 1.05~2.48) for subjects who underwent an appendectomy compared to comparison subjects. We found that among females, the adjusted HR of RA was 1.76 (95% CI = 1.04~2.96) for subjects who underwent an appendectomy compared to comparison subjects. However, there was no increased hazard of RA for males who underwent an appendectomy compared to comparison subjects. We concluded that female subjects who undergo an appendectomy have a higher risk of RA than comparison female subjects.

## Introduction

The vermiform appendix is part of gut-associated lymphoid tissue (GALT) which may be considered an immune organ in a natural environment [1,2]. Previous studies showed that changes in immune function after an appendectomy may be associated with a variety of diseases such as coeliac disease, ulcerative colitis, Crohn’s disease, *Clostridium difficile* infection, acute myocardial infraction, and pulmonary tuberculosis [3–10]. In addition, many studies reported a possible association of an appendectomy with rheumatoid arthritis (RA).

RA is a major autoimmune disease which primarily affects joints and is considered to be a chronic inflammatory disorder. Some case-control studies found that removal of lymphoid tissue can increase the risk of RA. However, some researchers reported that an appendectomy was not related to the subsequent risk of RA [11,12]. One study by Eftekharian and Mahdi [13] in Iran even concluded that an appendectomy was significantly and inversely associated with the risk of RA. Therefore, findings on the relationship between an appendectomy and RA remain inconsistent. Furthermore, all such studies were conducted in Western societies, and relevant studies of the relationship between an appendectomy and RA in Asian countries are still lacking. In addition, to date, most such studies have relied upon regional samples or on data from a few selected hospitals or subpopulations of subjects, and as such, do not permit unequivocal conclusions to be drawn.

The aim of this study was to investigate the relationship between an appendectomy and the subsequent risk of RA using a population-based dataset. In addition to its policy value on the question of optimal treatments for appendicitis, the study also contributes to the literature on this topic and enables cross-country comparisons.

## Methods

### Database

We retrieved data for this retrospective cohort study from the “Longitudinal Health Insurance Database 2005” (LHID2005). Taiwan implemented the National Health Insurance (NHI) program in 1995, and the coverage rate has been about 98.4% since its initiation. The LHID2000 includes registration files and original medical claims for 1,000,000 enrollees randomly selected from all enrollees listed in the 2000 Registry of Beneficiaries under the NHI program (*n* = 23.72 million) by the Taiwan National Health Research Institutes. Therefore, the LHID2000 allows researchers to longitudinally follow-up the utilization of medical services for these selected 1,000,000 enrollees since the beginning of the NHI in 1995. The LHID2000, which was open to the researchers in Taiwan, was available from the National Health Research Institutes (http://nhird.nhri.org.tw/date_01.html).

This study was exempt from full review by the Institutional Review Board of the National Defense Medical Center since the LHID2000 consists of de-identified secondary data released to the public for research purposes.

### Study sample

This study was designed to include a study cohort and a comparison cohort. For the study cohort, we first identified 5,412 subjects who had undergone an appendectomy (ICD-9-CM procedure code 470, 470.1, or 470.9) between January 1, 2002 and December 31, 2007 from the LHID2000. We then defined the date of the appendectomy as the index date for the study cohort. We further excluded subjects aged <18 years (*n* = 997). In addition, we excluded all subjects who had a history of severe forms of autoimmune diseases including ankylosing spondylitis (AS) (ICD-9-CM code 720 or 720.0), systemic lupus erythematosus (SLE) (ICD-9-CM code 710.0), and RA (ICD-9-CM code 714.0), systemic sclerosis (SSc) (ICD-9-CM code 710.1), Sjögren syndrome (SS) (ICD-9-CM code 710.2), or inflammatory myositis (ICD-9-CM code 359) before their index date (*n* = 121). As a result, 4,294 subjects who had undergone an appendectomy were included in the study cohort.

We likewise selected a comparison cohort from the remaining subjects in the registry of beneficiaries of the LHID2000. We randomly retrieved 12,882 subjects (three for every subject who underwent an appendectomy) to match the study cohort in terms of sex, age group (<40, 40~49, 50~59, 60~69, and >69 years), and the year of the index date using the SAS proc surveyselect program (SAS System for Windows, vers. 8.2, SAS Institute, Cary, NC). As for the study cohort, the year of the index date was the year in which they underwent an appendectomy. For the comparison cohort, the year of the index date was a matched year in which they had utilized medical care. In addition, we defined their first healthcare use occurring in the index year as their index date. We likewise ensured that none of the selected comparison subjects had a history of AS, SLE, RA, SSc, SS, or inflammatory myositis before their index date. Furthermore, we ensured that all selected comparison subjects did not undergo an appendectomy during the 5-year follow-up period. Ultimately, 17,176 sampled subjects were included in this study. We individually tracked each subject for a 5-year period from their index date to identify those who developed RA (ICD-9-CM code 714.0). In Taiwan, when a physician suspects that a subject has RA, he or she may give the subject a temporary diagnosis of RA in order to perform related clinical or lab tests for confirmation. The RA was usually diagnosed by specialists in rheumatology based upon clinical symptoms, radiographic changes, and identification of serum rheumatoid factor. If a subject had a confirmed RA diagnosis after these tests, he or she would receive routine treatment and have a second RA diagnosis in their next outpatient visit. On the other hand, if all feasible tests had excluded the possibility of AS, this subject would not receive a diagnosis of AS again. Therefore, in order to assure RA diagnostic validity, we only selected subjects who had received two or more RA diagnoses, with at least one being made by a specialist in rheumatology. Furthermore, we only included RA cases if they had been prescribed at least one type of disease-modifying antirheumatic drug (DMARD). We defined the date of first RA diagnosis as the RA onset date in this study.

### Statistical analysis

We used the SAS system for statistical analyses. Pearson Chi-squared tests were used to compare differences between subjects who underwent an appendectomy and comparison subjects in terms of monthly income (NT$0~15,840, NT$15,841~25,000, ≥NT$25,001; the average exchange rate in 2007 was US$1.00≈New Taiwan (NT)$30), geographical location (northern, central, eastern, and southern Taiwan), urbanization level of the subject’s residence (five levels with 1 being the most urbanized and 5 being the least), hypertension, diabetes, hyperlipidemia, pneumonia, urinary tract infections (UTI), alcohol abuse, and tobacco use disorder. We further used the Kaplan-Meier method and a log-rank test to examine the difference in 5-year RA-free survival rates between subjects who underwent an appendectomy and comparison subjects. Finally, we performed stratified Cox proportional hazard regressions (stratified by sex, age group, and the year of the index date) to calculate the hazard ratio (HR) and its corresponding 95% confidence interval (CI) for the subsequent development of RA during the 5-year follow-up period between subjects who underwent an appendectomy and comparison subjects. We also censored those subjects who died during that time (395 from the study cohort (9.2% of the subjects who underwent an appendectomy) and 1159 from the comparison cohort (9.0% of the comparison subjects)). We used a significance level of 0.05.

## Results

Table 1 presents the distributions of demographic characteristics and medical co-morbidities in terms of the presence or absence of an appendectomy. After being matched for sex, age group, and the year of the index date, there was a significant difference in monthly income (*p*<0.001) and geographic region (*p*<0.001) between subjects who underwent an appendectomy and comparison subjects. However, we observed no significant difference in urbanization level, hypertension, diabetes, hyperlipidemia, alcohol abuse, and tobacco use disorder between subjects who underwent an appendectomy and comparison subjects.

**Table 1:** Demographic characteristics of the sampled subjects (N = 17,176).

Variable	Subjects who underwent an appendectomy *N* = 4,294		Comparison subjects *N* = 12,882		*p* value
	Total no.	Column %	Total no.	Column %	
Male	2300	53.6	6900	53.6	1.000
Age group (years)					1.000
<40	1,430	33.3	4,290	33.3	
40~49	1,034	24.1	3,102	24.1	
50~59	781	18.2	2,343	18.2	
60~69	460	10.7	1,380	10.7	
>69	589	13.7	1,767	13.7	
Urbanization level					0.179
1 (most)	1,307	30.4	4,184	32.5	
2	1,215	28.3	3,546	27.5	
3	742	17.3	2,161	16.8	
4	577	13.4	1,659	12.9	
5 (least)	453	10.6	1,332	10.3	
Monthly income					<0.001
NT$0~15,840	1,578	36.8	5,487	42.6	
NT$15,841~25,000	1,614	37.6	3,907	30.3	
≥NT$25,001	1,102	25.7	3,488	27.1	
Geographic region					<0.001
Northern	2,024	47.1	6,344	49.3	
Central	964	22.5	2,999	23.3	
Southern	1,181	27.5	3,285	25.5	
Eastern	125	2.9	254	2.0	
Hypertension	891	20.8	2,552	19.8	0.183
Diabetes	486	11.3	1,348	10.5	0.110
Hyperlipidemia	762	17.8	2,198	17.1	0.305
Alcohol abuse	31	0.7	77	0.6	0.373
Tobacco use disorder	204	4.8	640	5.0	0.790

Note: The average exchange rate in 2007 was US$1.00≈New Taiwan (NT) $30.

**Fig 1:**
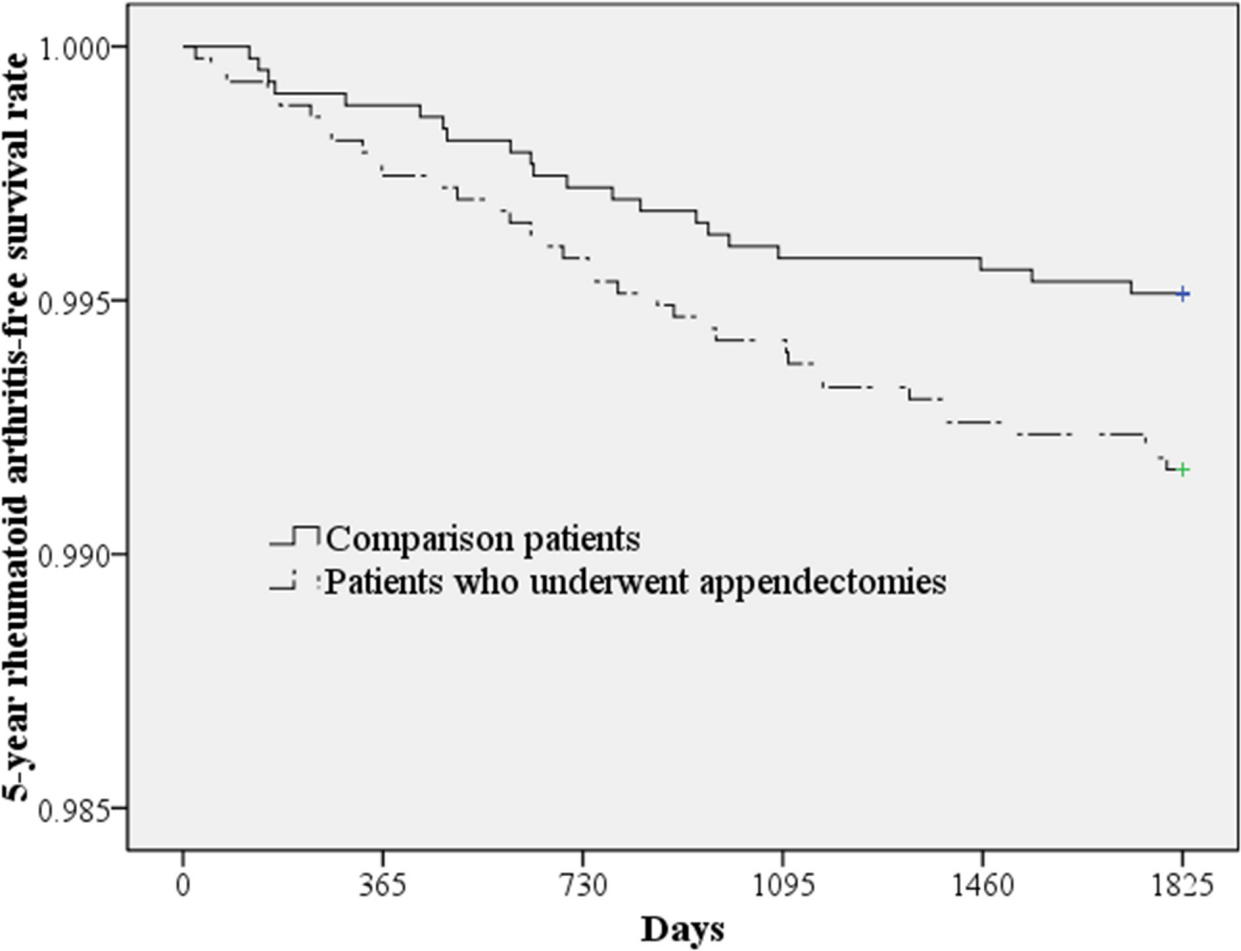
Five-year rheumatoid arthritis-free survival rates for those who underwent an appendectomy and comparison subjects.

Table 2 presents the incidence of RA during the 5-year follow-up period stratified by the presence of an appendectomy. Of sampled subjects, 93 (0.54%) received a diagnosis of RA during the 5-year follow-up period: 33 from the study cohort (0.77% of subjects who underwent an appendectomy) and 60 from the comparison cohort (0.47% of comparison subjects). The log-rank test suggests that subjects who underwent an appendectomy were more likely to have RA than comparison subjects (*p*<0.001). Fig 1 presents RA-free survival curves between subjects who underwent an appendectomy and comparison subjects.

**Table 2:** Crude and covariate-adjusted hazard ratios (HRs) for rheumatoid arthritis among the sampled subjects during the 5-year follow-up period.

Development of rheumatoid arthritis	Total sample *(N* = 17,176)		Subjects who underwent an appendectomy (*N* = 4,294)		Comparison subjects *(N* = 12,882)		
	No.	%	No.	%	No.		%
Five-year follow-up period							
Yes	93	0.54	33	0.77	60		0.47
Crude HR (95% CI)	-		1.66* (1.08~2.54)			1.00	
Adjusted ^a^ HR (95% CI)	-		1.61* (1.05~2.48)			1.00	

Notes: CI, confidence interval. The HR was calculated by a stratified Cox proportional hazard regression which was stratified by sex, age group, and the year of the index date.

^a^ Adjustments were made for subjects’ monthly income and geographic region.

* p<0.05.

The crude and adjusted HRs for the development of RA by cohort are shown in Table 2. The stratified Cox proportional analysis (stratified by age, sex, and the year of the index date) suggested that after censoring individuals who died during the follow-up period and adjusting for subjects’ monthly income and geographic region, the HR of RA during the 5-year follow-up period was 1.61 (95% CI = 1.05~2.48) for subjects who underwent an appendectomy compared to comparison subjects.

Table 3 analyzed the crude and adjusted HRs for the development of RA by cohort according to sex. We found that among females, the adjusted HR of RA was 1.76 (95% CI = 1.04~2.96) for subjects who underwent an appendectomy compared to comparison subjects. However, there was no increased hazard of RA for males who underwent an appendectomy compared to comparison subjects.

**Table 3:** Crude and covariate-adjusted hazard ratios (HRs) for rheumatoid arthritis among sampled subjects during the 5-year follow-up period by gender.

Development of rheumatoid arthritis	Gender							
	Male				Female			
	Subjects who underwent an appendectomy (*N* = 2,300) *n*, %		Comparison subjects (*N* = 6,900) *n*, %		Subjects who underwent an appendectomy (*N* = 1,994) *n*, %		Comparison subjects (*N* = 5,982) *n*, %	
Five-year follow-up period								
Yes	10	0.43	21	0.30	23	1.15	39	0.65
Crude HR (95% CI)	1.43 (0.67~3.04)		1.00		1.78* (1.06~2.98)		1.00	
Adjusted ^a^ HR (95% CI)	1.36 (0.64~2.91)		1.00		1.76* (1.04~2.96)		1.00	

Notes: CI, confidence interval; The HR was calculated by a stratified Cox proportional hazard regression which was stratified by sex, age group, and the year of the index date.

^a^ Adjustments were made for subjects’ monthly income and geographic region.

* p<0.05.

Table 4 further showed the crude and adjusted HRs for the development of RA on females according to reproductive age group (<40 vs. ≥40 years old). We found that of the females aged over 40 years old, the adjusted HR of RA was 1.96 (95% CI = 1.06~3.63) for subjects who underwent an appendectomy when compared to comparison subjects. However, of the females aged <40 years (reproductive age group), there was not an increased hazard of RA for subjects who underwent an appendectomy than comparison subjects.

**Table 4:** Crude and covariate-adjusted hazard ratios (HRs) for rheumatoid arthritis among sampled female subjects during the 5-year follow-up period by age.

Development of rheumatoid arthritis	Age							
	≥40 years old				<40 years old			
	Subjects who underwent an appendectomy (*N* = 857) *n*, %		Comparison subjects (*N* = 2,571) *n*, %		Subjects who underwent an appendectomy (*N* = 1,137) *n*, %		Comparison subjects (*N* = 3,411) *n*, %	
Five-year follow-up period								
Yes	17	2.07	27	1.08	6	0.53	12	0.35
Crude HR (95% CI)	1.91* (1.03~3.52)		1.00		1.50 (0.56~4.01)		1.00	
Adjusted a HR (95% CI)	1.96* (1.06~3.63)		1.00		1.38 (0.51~3.72)		1.00	

Notes: CI, confidence interval; The HR was calculated by a stratified Cox proportional hazard regression which was stratified by sex, age group, and the year of the index date.

^a^ Adjustments were made for subjects’ monthly income and geographic region.

* p<0.05.

## Discussion

Our population-based study found that the HR of RA during the 5-year follow-up period was 1.61 (95% CI = 1.05~2.48) for all subjects with an antecedent appendectomy compared to comparison subjects after censoring individuals who died during the follow-up period and adjusting for subjects’ monthly income. This finding is consistent with observations of a case-control study by Gottlieb et al. (1979) which found that RA subjects had a higher prevalence of a prior appendectomy than controls [11]. In addition, other research by Fernandez-Madrid et al. (1985) indicated that antecedent removal of lymphoid tissue from the appendix is a predisposing factor for RA [11,12].

However, some studies failed to establish an association between an appendectomy and RA. For example, Moens et al. examined 1524 RA subjects and 1194 subjects with osteoarthritis (OA) and found that RA was not associated with an antecedent appendectomy [14]. Another study by Linos et al. on 229 female subjects with RA and 458 controls found no association between RA and an antecedent appendectomy [15]. Furthermore, a study by ter Borg et al. observed that subjects with fibromyalgia had a higher prevalence of an antecedent appendectomy than did subjects with RA, but the difference was not statistically significant [16]. A study by Patel and Eastmond, on the contrary, even found that a preceding appendectomy had been performed in 18.5% of subjects with RA and 32.2% of subjects with OA (*p*<0.05) [17]. Another case-control study by Eftekharian and Mahdi also reported that an antecedent appendectomy may have a protective role against RA occurrence [13]. The inconsistent findings of prior studies could have been due to their cross-sectional designs, small sample sizes, and/or the lack of a comparable control group.

The mechanism between the relationship with an appendectomy and subsequent disease still remains unknown. Some evidence has shown that the appendix may be associated with substantial lymphatic tissue which is thought to play a specific role in immune function [18,19]. Therefore, the vermiform appendix can be regarded as a well-adapted safe house for maintaining normal gut bacteria, and it can provide support for bacterial growth [19]. In addition, evidence suggests that the appendix may be viewed as part of the immune system, which is important for life in a natural environment [1]. The removal of lymphoid tissue may change the immune function and result in chronic inflammatory diseases such as RA.

Additionally, this study found that female subjects who underwent an appendectomy had higher risk for RA than comparison subjects. However, there was no increased hazard of RA for males who underwent an appendectomy. Even though the actual mechanism still remains unclear, these results might be explained by the gender difference in the function of the immune system, because sex hormones might affect the function of lymphocytes and macrophages and further influence immune responses. Accordingly, females often showed more prevalent and greater severity of spontaneous autoimmune conditions compared to males. Therefore, it is plausible that females with no appendix (associated with substantial lymphatic tissue) might have higher risk of RA compared to males [20].

The strength of this study is based on the longitudinal database and large population size. Nevertheless, there are several limitations to our study. First, the LHID2000 provides no information on the level of education or habitual behaviors such as diet, smoking, and caffeine consumption which are potential risk factors for RA. Second, the LHID2000 contains no laboratory data, and thus, we were unable to explore the role of an appendectomy in the pathogenesis of RA. Third, the LHID2000 predominantly includes the ethnic Chinese population of Taiwan so the ability to generalize the results to other ethnic groups cannot be guaranteed.

In recent years, the literature has shown that non-operative treatments with antibiotics for acute appendicitis are safe and can reduce surgical risks and overall costs [21–23]. Consistently, our study provides evidence of a relationship between an appendectomy and an increased risk of RA. Therefore, we suggest that the optimal treatment for acute appendicitis be reconsidered. Furthermore, further studies are warranted to investigate clinical and experimental evidence to identify the role of an appendectomy in the pathogenesis of RA.
